# Equestrian Trauma in an Urban Environment: A Retrospective Analysis in a Level I Trauma Center

**DOI:** 10.7759/cureus.81609

**Published:** 2025-04-02

**Authors:** Elizabeth Swezey, Elisa Szydziak, L. D. George Angus, Vishes Mehta, Sara Cardozo-Stolberg

**Affiliations:** 1 Department of Surgery, Nassau University Medical Center, East Meadow, USA

**Keywords:** equestrian, horse, injuries, sports medicine, trauma, urban

## Abstract

Introduction: Equestrian trauma is recognized as a high-risk injury mechanism with the potential for significant morbidity and mortality. Although horse-related injuries are more frequently seen in a rural environment, they can also occur in urban settings. Urban physicians should be mindful of these potential cases.

Methods: A retrospective review was performed on all patients treated at Nassau University Medical Center, East Meadow, New York, for equestrian-related trauma from January 1, 2000, to December 31, 2024.

Results: Twenty-nine equestrian trauma patients ages 10-70 were included, with injuries observed in all six Abbreviated Injury Scale (AIS) body regions. Admission was required in 93% of patients. Intensive care unit (ICU) admission was required in 41% of the cases. Head and extremity injuries were the most common reasons for admission. Polytrauma patients were seen both in patients who fell from a horse and those who were kicked by a horse. The mean Injury Severity Score (ISS) was 6.9 and 11.8, respectively.

Conclusion: Both falling from a horse and being kicked by a horse have been shown to have a high risk of serious morbidity. Equestrian trauma can occur in urban environments. Urban physicians should be aware of injury patterns in equestrian trauma and maintain a high index of suspicion for serious injuries, as most patients will require admission and intervention.

## Introduction

Equestrianism is a popular sport across the world, being a source of both vigorous competition and a relaxing pastime for experienced and inexperienced riders alike. The Center for Disease Control estimates that 30 million people in the United States ride a horse each year, both for work and leisure-related activities [1]. There are 6.65 million horses in the United States alone, with an economic impact of $177 billion value added to the economy, and impacting 2.2 million jobs [2]. Horses are essential for farming, and mounted police can be seen in both rural and urban environments. Horseback riding classes and excursions are not uncommon. Competitive equestrian activities are numerous and range from mild events such as dressage to the more dangerous activities of horse racing and bronco riding.

Horses can be unpredictable, and equestrian-related accidents are increasingly being recognized as a cause of significant morbidity and mortality. The proportional incidence of equine-related injury is reported to surpass the incidence of injuries reported in many other leisurely activities, including riding a motorcycle, playing football, and skiing [3].

Equestrian-related trauma is typically encountered in suburban and rural environments where equestrian-related activities are concentrated. Injury patterns have been well described for equestrian trauma in a rural environment. Overall, various studies have shown that the thorax or the head is injured most commonly, although head and neck injuries remain a significant cause of mortality [1,3]. Upper-extremity fractures are the most common injury seen in younger patients, and rib fractures are the most common injury seen in older patients [3-5]. A full trauma workup is supported in older patients and patients who fall off a horse, as these are high-energy injuries that can result in a high incidence of morbidity [1,6-10]. Although these injuries are well described in rural and suburban environments, a review of the published literature reveals very little data relating to equestrian-related injuries occurring in an urban environment. The purpose of this study is to characterize the incidence and injury patterns of equestrian-related injuries that occur in the urban environment and compare these findings with those previously reported in the literature from suburban and rural environments. Even though equestrian trauma is more common in rural areas, it is important for physicians in urban settings to be familiar with injury patterns and demographics, as it is possible, and even likely, that they will encounter equestrian trauma and should maintain a high index of suspicion for serious injuries.

## Materials and methods

Study design and data collection

This is a retrospective observational study conducted at Nassau University Medical Center (NUMC), an urban Level I trauma center, and approval was obtained from the Institutional Review Board of Nassau Health Care Corporation at NUMC (approval number 21-392). The trauma registry was queried for all patients with equestrian-related mechanisms of injuries from January 1, 2000, to December 31, 2024, and selected for inclusion in the study. The identified cases were reviewed to ensure accuracy and relevance to the study objectives. Incomplete records were excluded from further analysis based on the nature and extent of missing data fields.

Demographic information, co-morbid conditions, pre-injury medications, mechanism of injury (MOI), vital signs, Abbreviated Injury Scale (AIS), Injury Severity Score (ISS), revised Trauma Score (rTS), injuries, Glasgow Coma Scale (GCS), admission disposition, hospital course, intensive care unit (ICU) length of stay, hospital length of stay (HLOS), ventilator days, operating room (OR) procedures, blood transfusion requirements in the first 24 hours, complications, disposition, and outcomes were extracted from the Trauma Registry and supplemented by direct review of the electronic medical record.

Study setting and population

NUMC is an urban 450-bed public safety net hospital located in Nassau County, New York, that serves a population of nearly 1.4 million people [11]. It is a Level I trauma center verified by the American College of Surgeons. The emergency department has approximately 75,000 visits, and the Trauma Center has approximately 1,700 admissions each year.

Data analysis

Descriptive statistics were used to summarize the demographic and clinical variables. Continuous variables were summarized by presenting mean and standard deviation. Categorical variables were summarized using frequency and percentages. Continuous variables were compared using the Wilcoxon rank-sum test. Fischer’s exact test was used to examine the association of categorical variables. A p-value of <0.05 was considered statistically significant. Patients were categorized into two categories (fall from horse vs. kicked by horse) to compare injury patterns, severity, and clinical characteristics, among others. Statistical analysis was performed using SAS 9.4 (SAS Institute Inc., Cary, North Carolina).

## Results

A total of 35 patients with equestrian-related trauma were identified from January 1, 2000, to December 31, 2024. Of those 35 patients, six were excluded due to incomplete records, leaving a study population of 29 patients. The demographic, injury, and hospitalization characteristics of the study population are presented in Table 1. Five patients were kicked by a horse, and 24 patients fell from a horse. Of our patients, 86% were female. The mean ages were 33.2 for the patients who fell from a horse and 50.8 for those kicked by a horse. All patients lived. No statistically significant differences were found between the two groups, including AIS/ISS, GCS, or need for OR or ICU admission. Head and neck injuries were the most common injuries overall (52%). Extremity injuries represented 34% of injuries, and thoracic injuries accounted for 31%. Abdominal injuries were present in 17% of patients. The GCS was 15 in 28 out of 29 patients, and one patient had a GCS of 12.

**Table 1:** Comparison of equestrian injuries and patient characteristics (fall from horse vs. kicked by horse) Mean ± SD are presented for continuous variables, with the median in parentheses. Statistical comparisons were performed using the Wilcoxon rank-sum test (*) for continuous variables and Fisher’s exact test (**) for categorical variables.

Variable	Unit	Fall from horse (n = 24)	Kicked by horse (n = 5)	p-value
Age* (years)	mean +SD (median)	33.2 ± 19.3 (26)	50.8 ± 8.6 (50)	0.0841
Sex** (female)	n (%)	21 (87.5)	4 (80.0)	0.5526
Lived**	n (%)	24 (100.0)	5 (100.0)	NA
Intubated**	n (%)	0 (0.0)	1 (20.0)	0.1724
OR admission**	n (%)	5 (20.8)	2 (40.0)	0.5688
ICU admission**	n (%)	10 (41.7)	2 (40.0)	1.0000
Abbreviated Injury Scale (AIS)				
AIS: head/neck injury**	n (%)	14 (58.3)	1 (20.0)	0.1686
AIS: face injury**	n (%)	1 (4.2)	0 (0.0)	1.0000
AIS: chest injury**	n (%)	6 (25.0)	3 (60.0)	0.2872
AIS: abdominal injury**	n (%)	3 (12.5)	2 (40.0)	0.1947
AIS: pelvis/extremities injury**	n (%)	8 (33.3)	2 (40.0)	1.0000
AIS: external injury**	n (%)	9 (37.5)	0 (00.0)	0.1528
Activation level **				0.5510
Level 1	n (%)	9 (37.5)	3 (60.0)	
Level 2	n (%)	7 (29.2)	0 (0.0)	
Level 3	n (%)	5 (20.8)	1 (20.0)	
Not called	n (%)	3 (12.5)	1 (20.0)	
Injury Severity Score (ISS)*	Mean +SD (median)	6.9 ± 4.9 (4.5)	11.8 ± 8.0 (9.0)	0.1591
Glasgow Coma Scale (GCS)*	Mean +SD (median)	14.9 ± 0.6 (15)	15.0 ± 0 (15)	0.7177

Table 2 presents the primary injuries by mechanism of action. For those patients who were kicked by a horse, lower-extremity fractures, lower-extremity compartment syndrome, and spinal fractures were seen, as well as two patients with multiple injuries. Injuries for patients who fell from a horse included head injuries; bony injuries to the extremities, ribs, and spine; pelvic fractures; facial injuries; and patients with multiple injuries.

**Table 2:** Primary injuries of equestrian injuries by mechanism of injury

Primary injury	Fall from horse (n)	Kicked by horse (n)	Total (n)
Concussion	6	0	6
Polytrauma	3	2	5
Spine fractures	2	1	3
Upper-extremity fractures	3	0	3
Lower-extremity fractures	1	1	2
Pelvic ring injury	2	0	2
Rib fractures	2	0	2
Superficial head injury	2	0	2
Clavicle fracture	1	0	1
Facial fractures	1	0	1
Facial injury	1	0	1
Lower-extremity compartment syndrome	0	1	1
Total	24	5	29

## Discussion

Horses can range in weight from 750 pounds to 2000 pounds and, depending on the breed, can reach speeds of 40-50 miles per hour [12,13]. Falling from a horse can lead to significant morbidity, particularly if the horse is moving at speed. The forces generated by a fall from a horse moving at high speeds and jumping have been described as comparable to losing control of a motorcycle [1]. A horse can kick at speeds of 220 miles per hour, generating over 8,000 Newtons of force (2000 pounds per square inch), irrespective of whether or not the kick was provoked [14,15]. To put that in perspective, a heavyweight boxer’s punch delivers 1420 pounds per square inch of force [16]. In an observational study of children under the age of 15, the mean modified injury severity scores of injured riders were higher only in pedestrian-struck patients [17].

As shown in Table 1, the ISS of our equestrian trauma patients had a mean of 6.9 for a fall from a horse and 11.8 for the patients kicked by a horse, although this difference in ISS did not reach statistical significance. The ISS was in the mild to moderate range for 90% of our patients, with only three patients having a severe ISS. Admission was indicated for 93% of our patients, with 41% requiring ICU admission and one patient requiring mechanical ventilation. Seven (24%) patients required operative intervention. Six of these were for the repair of a bone fracture, and one was for the management of a fasciotomy wound. One patient exhibited a moderate traumatic brain injury, but all others presented with a GCS of 15. None of our findings reached statistical significance based on the mechanism of injury, and the mortality for our study population was zero.

Mutore et al.’s review of the National Trauma Data Bank for equestrian trauma over a 10-year period included 24,791 patients [1]. A mild to moderate ISS was seen in 76.9% of their patients, which is consistent with our findings. They had similar admission rates (88.1%) but much lower ICU admission rates (28.3%). A study from rural Scotland of 162 patients had an even lower ICU admission rate of 8.6% [7]. Acton et al., reviewing emergency department treatment of equestrian trauma in the United States, had an admission rate of 12.3%, although this may be because it captured a wider range of patients treated in the emergency department after equestrian trauma, not just those requiring trauma team evaluation [8]. Studies by Mutore et al. and Dick et al. showed lower rates of operative intervention: 9.8% and 8.3% [1,7]. Mutore et al.'s rates of traumatic brain injury were also consistent with ours, as 88% of their patients had a GCS of 13-15 [1].

Different studies have shown that the most common injuries were in the thoracic region or the head and neck region, although head and neck injuries lead to the highest mortality [1,3]. The primary injuries requiring admission in our study were broken down by mechanism of injury in Table 2. Head and neck injuries were the most common overall. In patients who fell from a horse, injuries requiring admission included six patients with extremity fractures, eight patients with head and neck injuries, and three patients who sustained multiple injuries. While injuries were more focal in patients who were kicked by a horse (two lower-extremity injuries and one spinal fracture), there were two patients who presented with multiple injuries.

Our study population was 86% female (Table 1). Previous studies also note a predominance of female patients. A study of 162 patients by Dick et al. noted 74.7% of their patients were female, and another study of 144 patients by Liaw et al. noted 86% of their patients were female [7,18]. Although these findings may be affected by the small sample size, 2021 demographics on equestrian participants show that 71.7% of equestrians in the United States are female [19].

Equestrianism, which is often associated with rural environments, can be found in an urban setting. A review of the literature shows a plethora of studies available on equestrian trauma, though none reviewing it in an urban setting. The majority of equestrian trauma requiring trauma team activation requires admission to the hospital. Dick et al. had 26.5% of their patients present with multiple injuries, and our study concurred with 31% presenting with multiple injuries [7]. Horseback riding has higher rates of injury per activity hours than many other high-risk activities, including skiing and riding a motorcycle, and is a high-energy mechanism of injury [1]. Our rates of ICU admission were almost double those reported nationally [1]. Although our small sample size leaves us unable to make statistically significant conclusions on injury patterns across demographics, the urban physician must be aware of these trends and the risk for serious injury that patients presenting after equestrian trauma have.

Limitations

This study provides valuable insight into equestrian trauma in an urban setting, and we ensure a comprehensive assessment of injury patterns and outcomes by using trauma registry data spanning more than two decades. However, this study has limitations. First, the sample size is small, which limits the ability to detect statistically significant differences between injury patterns and patient outcomes based on the mechanism of injury. The small sample size also affects the generalizability of our findings to a larger equestrian trauma population. Second, this study is retrospective in nature, relying on data from the trauma registry, which may have incomplete documentation of injury details. Future studies with larger, multicenter datasets and prospective follow-up are needed to further investigate equestrian trauma patterns and outcomes.

## Conclusions

Equestrian trauma is not unheard of in an urban environment, and the majority require hospital admission. In addition, close to 40% required ICU admission, and 24% required operative intervention. This study shows that even physicians in urban settings should be familiar with equestrian trauma. Equestrian-related trauma is a high-energy mechanism of action and can result in serious injury requiring admission with likely intervention. Even in an urban setting, the physician involved in trauma care should have a high index of suspicion for serious injury and polytrauma, both in patients who fall from a horse and in patients kicked by a horse.
